# An insight into the biochemistry of inborn errors of metabolism for a clinical neurologist

**DOI:** 10.4103/0972-2327.41873

**Published:** 2008

**Authors:** Rita Christopher, Bindu P. Sankaran

**Affiliations:** Department of Neurochemistry, National Institute of Mental Health and Neuro Sciences, Post box 2900, Bangalore-560 029, India; 1 Department of Neurology, National Institute of Mental Health and Neuro Sciences, Post box 2900, Bangalore-560 029, India

**Keywords:** Biochemical tests, diagnosis, inborn errors

## Abstract

Neurological dysfunction is an important manifestation of inherited metabolic disorders. Although these are more common in childhood, adult onset forms with a different clinical presentation are often encountered. Recent advances in the diagnosis and treatment of these conditions have substantially improved the outcome in many of these conditions. This makes it essential that the practicing physician be familiar with the clinical presentation and diagnosis of these disorders. For the evaluation of a patient with a possible inborn error of metabolism, simple screening tests may aid in the diagnosis and provide direction for more comprehensive laboratory analysis. In this review, we present a practical approach to diagnosis of neurometabolic disorders. Establishing a specific diagnosis in these disorders will enable the clinician in offering a definitive long-term treatment, prognosis and genetic counselling.

## Introduction

Metabolic disorders caused by genetic mutations resulting in enzyme deficiencies in an intermediary metabolic pathway constitute a wide spectrum of diseases in clinical practice. The term ‘inborn errors of metabolism’ was introduced by Sir Archibald Garrod at the beginning of the twentieth century.[1] Since then, a great variety of hereditary metabolic disorders have been identified, and there has been a phenomenal increase in the knowledge regarding these disorders.[2] Although individually rare, they are collectively numerous. Their number, complexity and varied clinical presentation present a formidable challenge to the clinician. Yet, in many cases, prevention of death or permanent neurological sequele in patients with these disorders is dependent on early diagnosis and institution of appropriate therapy. In addition to comprehensive clinical assessment, imaging studies, electrophysiological investigations and histopathological information from biopsies, which help in establishing the distribution and type of abnormality, biochemical studies are required in many cases to confirm the diagnosis.

Over one-third of the inherited metabolic disorders are characterized by the central nervous system involvement and neurological symptoms are the presenting and the most prominent clinical problems associated with them. Among the neurometabolic disorders, there are particularly five common neurological presentations: chronic encephalopathy, acute encephalopathy, movement disorder, myopathy and psychiatric or behavioral abnormalities.[3]

## Chronic Encephalopathy

**Figure 1:**
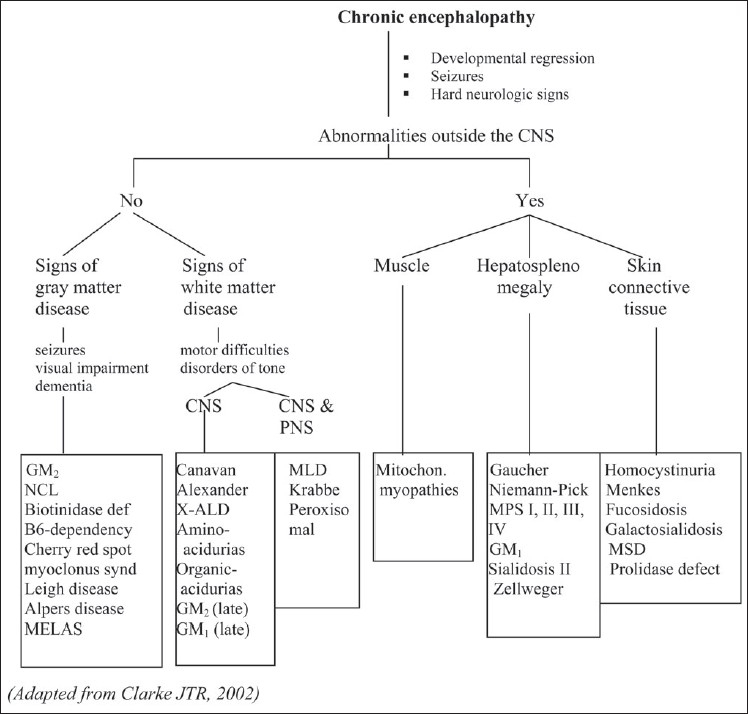
An approach to inherited metabolic disorders with chronic encephalopathy.[3] GM2: GM2 gangliosidosis, GM1: GM1 gangliosidosis, NCL: neuronal ceroid lipofuscinosis, MELAS: mitochondrial encephalopathy lactic acidosis syndrome, X-ALD: X-adrenoleukodystrophy, MLD: metachromatic leukodystrophy, MPS: mucopolysaccharidosis, MSD: multiple sulfatase deficiency

Of all neurological problems that occur in patients with inherited neurometabolic disorders, developmental delay or psychomotor retardation is the most common. The cognitive disabilities caused by these disorders tend to be at a global level, affecting all spheres of development to some extent. They are usually progressive and associated with other objective evidences of neurological dysfunction such as disorders of muscle tone, impairment of special senses, seizures, pyramidal tract signs, evidences of extrapyramidal deficits or cranial nerve deficits. Routine screening for inborn errors of metabolism in children with developmental delay has a diagnostic yield of approximately 1% that can increase to 5% in specific situations such as in the case of relatively homogenous and isolated populations or if there are clinical indicators.[4] A general approach to the evaluation of neurometabolic causes of chronic encephalopathy[3] is presented in Figure 1.

## Acute Encephalopathy

Acute encephalopathy presents in a number of neurometabolic disorders, particularly in children.[5] Major inherited metabolic disorders that can cause acute encephalopathy include disorders of amino acid metabolism (maple syrup urine disease, urea cycle disorders, and nonketotic hyperglycinemia), organic acidemias, fatty acid oxidation defects and mitochondrial respiratory chain defects. Because of the importance of identifying treatable neurometabolic disorders, initial investigation of any patient presenting clinically in a stuperous or obtunded state should not be delayed. Table 1 gives a summary of the expected results of the initial laboratory studies in various neurometabolic disorders presenting as acute encephalopathy.[3]

**Table 1:** Differential diagnosis of neurometabolic disorders presenting as acute encephalopathy

Biochemical investigation	Urea cycle disorders	Maple syrup urine disease	Organic acidurias	Fatty acid oxidation defects	Nonketotic hyperglycinemia
Metabolic acidosis	not present	variably present	present +++	variably present	not present
Plasma glucose	normal	normal or decreased ↓	decreased ↓↓	decreased ↓↓↓	normal
Urinary ketones	normal	elevated ↑↑	elevated ↑↑	absent	normal
Plasma ammonium	elevated ↑↑↑	normal	elevated ↑↑	elevated ↑	normal
Plasma lactate	normal	normal	elevated ↑	variable	normal
Liver function tests	normal	normal	normal	liver enzymes elevated	normal
Plasma amino acids	abnormal	↑ branched chain amino acids	↑ glycine	normal	↑ glycine
Urinary organic acids	normal	abnormal	abnormal	abnormal	normal
Plasma carnitine	normal	normal	abnormal	abnormal	normal

## Ataxia and Extrapyramidal Movement Disorders

Extrapyramidal movement disorders in patients with inherited neurometabolic disorders are almost always associated with neurological signs referable to other parts of the nervous system. Progressive ataxia may be the presenting symptom in many late-onset lysosomal storage disorders (late-onset metachromatic leukodystrophy, Krabbe disease, galactosialidosis, GM_2_ gangliosidosis and Niemann-Pick type C), abetalipoproteinemia, mitochondrial electron transport chain defects, neuronal ceroid lipofuscinosis, Refsum disease and Hartnup disease.[6] L-2-Hydroxyglutaric aciduria is a newly identified metabolic disorder, which can present with ataxia and macrocephaly in adulthood.[7,8] Differentiation from nonmetabolic hereditary ataxias is usually possible due to the presence of other neurological signs such as psychomotor retardation and evidence of nonneurological involvement with the disease. All patients with recurrent episodes of ataxia separated by symptom-free intervals should be investigated for a possible metabolic disorder. Intermittent ataxia is a common manifestation of metabolic decompensation in urea cycle enzyme defects, organic acidopathies, maple syrup urine disease variants, and pyruvate dehydrogenase deficiency.[9] The simple biochemical differentiation of these defects,[3] is given in Table 2.

**Table 2:** Diagnostic approach for inherited neurometabolic disorders presenting as recurrent attacks of ataxia

Associated biochemical feature	Most frequent diagnosis	Differential diagnosis
Ketoacidosis	Late-onset maple syrup urine disease, methylmalonic aciduria, propionic aciduria, isovaleric aciduria	Diabetes mellitus
Hyperammonemia	Urea cycle defects (ornithine transcarbamoylase deficiency, argininosuccinic aciduria)	Intoxications, encephalitis
Hyperlactacidemia		
Normal lactate/pyruvate, no ketosis, High lactate/pyruvate, ketosis	Pyruvate dehydrogenase deficiency, Multiple carboxylase defect mitochondrial respiratory chain defects	Migraine, cerebellitis, acetazolamide-responsive ataxia polymyoclonia
Generalized aminoaciduria	Hartnup disease	-

Extrapyramidal signs are an important manifestation of childhood neurometabolic disorders.[10,11] Glutaric aciduria should be high on priority in the differential diagnosis in children with acute profound dyskinesia or subacute motor delay accompanied by severe choreoathetosis and dystonia.[12] In methylmalonic and propionic acidemias, there can be late-onset extra pyramidal disease.[13] Choreoathetosis is characteristically observed in Lesch-Nyhan syndrome and should be a diagnostic consideration in boys who present with predominant extrapyramidal signs in the second year of life. In addition, it may sometimes present with athetosis without cognitive impairment or behavioral abnormalities in adulthood.[14] Parkinsonism and dystonia are the prominent features in many patients with Wilson disease. Dystonia of the extremities growing worse during the course of the day with normal intellect and a dramatic response to treatment with L-dopa is characteristic of Segawa syndrome,[15] Lysosomal storage disorders that can present with dystonia include Niemann-Pick disease type C,[16,17] Gaucher disease type 3, GM2 gangliosidosis[18,19] and GM1 gangliosidosis.[20,21]

## Myopathy

Inherited metabolic disorders presenting as myopathy are generally the result of defects in energy metabolism.[22] They consist of the following three categories: progressive muscle weakness, exercise intolerance with cramps and myoglobinuria and myopathy as a manifestation of multisystem disease.

Progressive muscle weakness is a characteristic feature in Pompe disease (Glycogen storage disorder (GSD) II). Progressive skeletal myopathy, occasionally involving the heart, may be a major problem in patients with Cori/Forbes disease (GSD III). Exercise intolerance with or without myoglobinuria is typical of a number of inherited defects of glycolysis, such as muscle phosphofructokinase deficiency (GSD VII), carnitine palmitoyl transferase II (CPT II) defect, myoadenylate deaminase deficiency, long-chain acyl–coenzyme A (CoA) dehydrogenase (LCAD) and short-chain hydroxyacyl-CoA dehydrogenase (SCHAD) defects. The biochemical differentiation of these defects is given in Table 3. The ischemic forearm exercise test is useful in the evaluation of these patients.[23] This test investigates the production of muscle energy through anaerobic glycolysis and the activation of the purine nucleotide cycle, which involves irreversible deamination of adenosine-5′-monophosphate (AMP) to inosine-5′-monophosphate (IMP), with ammonia production.

**Table 3:** Biochemical differentiation of inherited metabolic disorders presenting as muscle cramps[3]

Disease	Results of ischemic forearm exercise test	Other biochemical features	Confirmatory test
McArdle disease (GSD V)	Normal pretest lactate and no increase post-test	Elevated CPK myoglobinuria	Deficiency of phosphorylase in muscle
Phosphofructokinase (PFK) deficiency	Excessive increase of ammonium	Myoglobinuria, hyperuricemia, elevated CPK	Deficiency of PFK in muscle
Phosphoglycerate kinase (PGK) deficiency	Normal response	–	Deficiency of PGK in erythrocytes
Phosphoglycerate mutase (PGAM) deficiency	Excessive increase of ammonium	Myoglobinuria, hyperuricemia, elevated CPK	Deficiency of PGAM in muscle
Lactate dehydrogenase (LDH) defect	No lactic acidosis, but marked hyperpyruvic} acidemia during test	Myoglobinuria, hyperuricemia, elevated CPK	Deficiency of LDH-M subunit in erythrocytes
Carnitine palmitoyl transferase II (CPT II) deficiency	Normal lactate and ammonium response, but increased CPK	Increased CPK during fasting, myoglobinuria	Deficiency of CPT II in fibroblasts
Long-chain acyl-CoA dehydrogenase defect (LCAD)	Normal lactate and ammonium response, but increased CPK	Decreased plasma carnitine	Characteristic acylcarnitine profile, deficiency of LCAD in fibroblasts
Short-chain hydroxyacyl-CoA dehydrogenase (SCHAD) defect	Normal response	Myoglobinuria	Characteristic acylcarnitine profile Deficiency of SCHAD in fibroblasts
Myoadenylate deaminase deficiency	Normal lactate response, no increase in ammonium	Elevated CPK in 50%	Deficiency of myoadenylate deaminase in muscle

Progressive myopathy is also often the principal manifestation of mitochondrial electron transport chain defects, although other systems are invariably involved. Most are associated with persistent lactic acidosis, although lactate levels are generally not more than 10 mmol/l except during acute metabolic decompensation.

## Psychiatric problems

In many late-onset neurometabolic disorders, the first indication of the onset of disease may be behavioral or personality changes.[24–28] Severe irritability, impulsivity, aggressiveness and hyperactivity are also more common among children with mental retardation caused by a metabolic disorder. Some of the neurometabolic disorders presenting with behavioral problems and their diagnostic tests are given in Table 4.

**Table 4:** Inherited metabolic disorders characterised by psychiatric or behavioral abnormalities[3]

Disease	Psychiatric/ Behavioral Abnormality	Diagnostic laboratory tests
Late-onset metachromatic leukodystrophy	Anxiety, emotional lability, disorganised thinking, poor memory, psychosis	Leukocyte arylsulfatase A ↓ Mutation studies
Late-onset GM_2_ gangliosidoses	Acute psychosis, agitation hallucinations	Leukocyte β-hexosaminidase A ↓
X-Linked adrenoleukodystrophy	Social withdrawal, irritability, obsessional behavior, rigidity	Plasma very-long-chain fatty acids ↑
Lesch-Nyhan syndrome	Self-mutilatory behavior	Serum uric acid ↑, urine uric acid/creatinine ratio ↑
Wilson disease	Anxiety, depression, mania schizophrenia, antisocial behavior	Serum copper ↓, serum ceruloplasmin ↓ urine copper ↑, hepatic copper ↑
Acute porphyrias	Anxiety, depression, paranoia, restlessness	Urine porphobilinogen ↑
Sanfilippo disease (MPS III)	Hyperactivity, impulsiveness, aggressiveness, sleeplessness	Urine mucopolysaccharides↑ heparan sulfate present; Assay of relevant enzymes
Hunter disease (MPS II)	Hyperactivity, impulsiveness, aggressiveness, sleeplessness	Urine mucopolysaccharides ↑ Heparan sulfate, dermatan sulfate present
Urea cycle disorders	Periodic acute agitation, hallucination, anxiety	Plasma ammonium ↑, abnormal plasma amino acids

## Biochemical investigations

The initial biochemical investigation of a suspected inherited neurometabolic disease should include the following tests: blood glucose, liver function tests, blood gases and electrolytes, lactate, ammonium, creatine phosphokinase (CPK), lactate dehydrogenase (LDH), plasma and urine amino acid analysis (screening by thin-layer chromatography will fulfil most requirements, quantitative amino acid analysis if abnormalities are found), urine reducing compounds, ketones, mucopolysaccharides and oligosaccharide screening test. Further detailed investigations such as urinary organic acids, plasma carnitine and acyl carnitine profile, plasma very-long-chain fatty acids, red cell plasmalogens, plasma and urinary pipecolic acid, serum copper, ceruloplasmin, 24-h urinary copper, serum uric acid, urine uric acid/creatinine ratio, urine myoglobin, ischemic forearm exercise test, blood lipid profile and lipoprotein electrophoresis, cerebrospinal fluid (CSF) lactate and neurotransmitter studies, enzyme studies and molecular genetic studies could be carried out, if indicated, on the basis of the results of initial tests as well as the clinical findings.

## Glucose

Glucose is of essential, fundamental importance for brain metabolism. The major source of glucose to the brain is the blood supply; and, a fall in the blood glucose level may lead to severe encephalopathy. Hypoglycemia (blood glucose, <45 mg/dl; in neonates, <30 mg/dl), is a common nonspecific problem in severely ill neonates and young children irrespective of the illness. In adults, there are varied causes of hypoglycemia but are noted most often in diabetic patients and are usually secondary to changes in medication or overdoses, infection or changes in diet or activity. Other causes include islet cell/extrapancreatic tumors, adrenal insufficiency, hypopituitarism, severe hepatic dysfunction, sepsis or starvation. The most frequent causes of persisting neonatal hypoglycemia are hormonal disturbances, e.g., hyperinsulinism or hypopituitarism, or regulatory disturbances (e.g., ketotic hypoglycemia or glycogen storage disorders). Various etiologies of genetic hypoglycemias are classified according to the time of manifestation [Table 5].[29] The laboratory investigations during symptomatic hypoglycemia should include blood counts, C-reactive protein (CRP), liver function tests, creatine kinase (CK), uric acid, triglycerides, blood gases and electrolytes, lactate, and ammonia, ketones in urine, organic acids (in first urine sample after hypoglycemia), plasma amino acids, carnitine and acylcarnitines, plasma insulin, C-peptide, glucagons, cortisol, IGF-1 and isoelectric focussing for transferrin (if indicated).

**Table 5:** Etiology of genetic hypoglycemias[5]

*Immediate postprandial (within several minutes after feeding)*	
•	Hyperinsulinism (Plasma insulin >3 mU/l when glucose is <40 mg/dl)
	Sulphonylurea receptor (SUR 1) defect
	Inward-rectifying potassium channel (Kir.2) defect
	Glutamate dehydrogenase (GLUD 1) deficiency
	Glucokinase (GK) gene defects
	Short-chain 3-hydroxyacyl-CoA dehydrogenase (SCHAD)
	CDG types Ia and Ib
	Usher Ic (contiguous gene syndrome)
	Beckwith-Wiedemann syndrome
	Sotos' syndrome
	Perlman's syndrome
*A few hours after meal (3-4 h)*	
•	Glycogenosis I and III
•	Glycogen synthase deficiency
•	Respiratory chain disorders
*Fasting period*	
•	Fatty acid oxidation disorders
•	Fructose-1,6-bisphosphatase deficiency
•	Ketogenesis and ketolytic defects
•	Respiratory chain disorders
•	Fanconi-Bickel syndrome
•	Endocrinological causes
	Growth hormone (GH) deficiency
	Insulin-like growth factor-1 (IGF-1) defects
	Glucagon deficiency
	Adrenal steroid disorders
*Others*	
•	Hereditary fructose intolerance (induced by fructose ingestion)
•	Galactosemia (induced by galactose ingestion)
•	Tyrosinemia type I
•	Glucose transporter GLUT 1 defect (↓ CSF glucose only)

**Figure 2:**
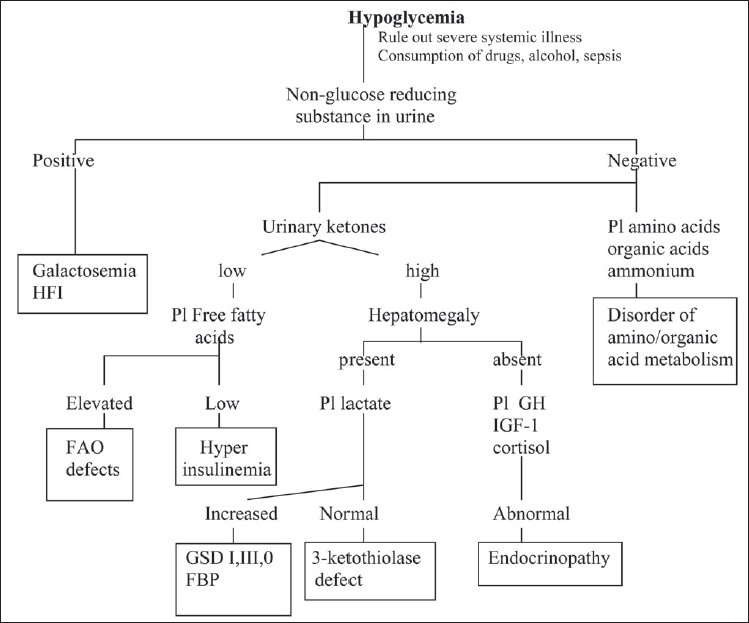
Biochemical evaluation of hypoglycaemia Pl: Plasma, HFI: Hereditary fructose intolerance, FAO: Fatty acid oxidation, GSD: Glycogen storage disorder, FBP: Fructose-1,6-bisphosphatase deficiency, GH: Growth hormone, IGF-1: insulin-like growth factor-1

Figure 2 presents an overview of a biochemical approach to the diagnosis of hypoglycemia, which focuses primarily on the disease caused by inherited neurometabolic disorders. The presence of non-glucose-reducing substances in the urine is characteristic of untreated classical galactosemia and hereditary fructose intolerance. This can be determined at the bedside by testing a few drops of urine with Benedict's test and the glucose strip (Uristix). A positive Benedict's test with a negative test with uristix for glucose indicates that the reducing substance is not glucose. Patients with galactosemia show other evidences of hepatocellular dysfunction and hereditary fructose intolerance is associated with marked lactic acidosis.

The normal physiological response to decreased glucose production is increased mitochondrial fatty acid β-oxidation and the production of ketones. Accordingly, increased urinary ketones provide an indirect evidence of whether hypoglycemia is the result of inadequate production or overutilization of glucose. In older infants, children and adults, the absence of ketones in urine is usually a strong indication of increased glucose utilization. Increased glucose utilization (hypoketotic hypoglycemia) occurs as a result of either hyperinsulinism or of primary or secondary defect in fatty acid oxidation. The two situations are distinguishable by measuring plasma free fatty acid levels. One of the physiological effects of insulin is inhibition of hormone-sensitive lipase in adipose tissue. Low free fatty acid levels during hypoglycemia are a strong indication of abnormally elevated insulin levels. The timing of the hypoglycemia and other laboratory findings render differentiation of this condition relatively simple. Hyperinsulinemic hypoglycemia occurs due to insulin hypersecretion by the islets of Langerhans. Focal islet cell hyperplasia is associated with mutation of sulfonylurea receptor (SUR1) or inwardly rectifying potassium channel (Kir.2) genes. A few cases of syndromic hyperinsulinemia such as hyperinsulinism associated with Usher syndrome type Ic, congenital disorder of glycosylation (CDG) Ia or Ib, Beckwith Wiedemann's syndrome, Perlman's or Sotos' syndrome have been described. Infants with mutations in glutamate dehydrogenase gene (GLUD I) present with recurrent hypoketotic hypoglycemia, elevated plasma insulin and persistent hyperammonemia.

Most hypoglycemias associated with permanent hepatomegaly occur due to an inherited metabolic disorder.[30] All conditions, acquired or inherited, associated with severe liver failure can result in severe hypoglycemia, which appears after 2–3 h of fasting and manifests with moderate lactic acidosis and no ketosis. When hepatomegaly is the most prominent feature without hepatic insufficiency, deficiencies of glucose-6-phosphatase (GSD I), fructose 1,6-bisphosphastase (FBP), glycogen debrancher (GSD III) or glycogen synthase (GSD 0) should be considered as the most probable diagnosis. In GSD I, laboratory examination typically shows lactic acidosis, hyperuricemia, hypertriglyceridemia and hypophosphatemia. Hypoglycemia is characteristically unresponsive to glucagon administration. A distinguishing feature of this disorder is a significant increase in plasma lactate in response to glucagon. In FBP deficiency, however, the response to glucagon is preserved. An oral glucose test can differentiate GSD I from GSD III. A moderate increase in blood lactate is observed in GSD III, whereas blood lactate drops precipitously in GSD I. The rare glycogen synthase deficiency presents with fasting hypoglycemia, ketosis and postprandial hyperlactacidemia. A definitive diagnosis of these disorders requires measurement of the relevant enzymes. Respiratory chain disorders can present with hepatic failure and hypoglycemia.

Hypoglycemia is a prominent secondary metabolic phenomenon in all mitochondrial fatty acid β-oxidation defects. The diagnosis can be confirmed by demonstrating the presence of high concentration of C6–C10 dicarboxylic acids (adipic, suberic and sebacic acids) and the characteristic acylcarnitines in plasma during acute decompensation. Fasting hypoglycemia and marked hepatomegaly associated with early-onset renal tubular dysfunction characterised by polyuria, hypophosphatemic rickets, hyperchloremic metabolic acidosis, and severe growth retardation is typical of Fanconi-Bickel syndrome. This condition is caused by mutation in GLUT 2 gene coding for the hepatic-type glucose transporter.

Patients with genetic endocrine disorders due to defects such as growth hormone (GH) gene deletion, mutation in GH-releasing hormone (GHRH) receptors, insulin-like growth factor-1 (IGF-1) defects or malformations of the hypothalamic area leading to multiple pituitary hormone deficiencies can present with fasting hypoglycemia. Hypoglycemic responsiveness to glucagon is variable—mild, absent or dramatic—the latter response being similar to that observed in hyperinsulinism. Hypoglycemia associated with isolated adrenocorticotrophic hormone (ACTH) deficiency is rare. Glucagon deficiency can also be associated with hypoglycemia.

## Blood Gases and Acid-base Profile

**Figure 3:**
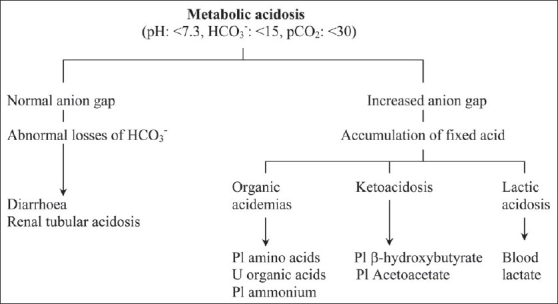
Evaluation of metabolic acidosis

**Figure 4:**
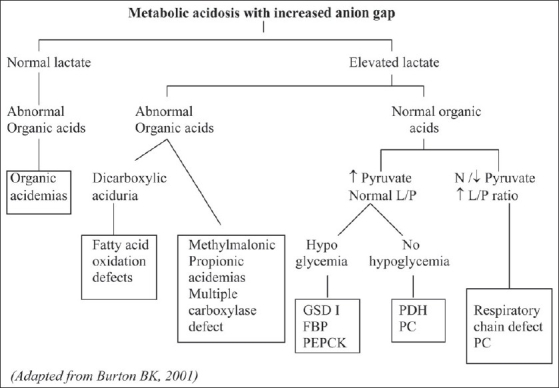
Evaluation of metabolic acidosis with increased anion gap **L/P:** Lactate/pyruvate ratio, N: Normal, ↓: Decreased, ↑: Increased, GSD I: glycogen storage disorder type I, FBP: fructose-1, 6-bisphosphatase deficiency, PEPCK: phosphoenolpyruvate carboxykinase deficiency, PDH: Pyruvate dehydrogense deficiency, PC: pyruvate carboxylase deficiency

The second most important laboratory feature of many inherited neurometabolic disorders during episodes of illness is metabolic acidosis that is demonstrated by measuring arterial blood gases and bicarbonate. An increased anion gap (>16) is observed in many of these disorders due to the accumulation of fixed acids such lactic acid, ketoacids and other organic acids [Figure 3]. Diagnostically, identifying the unmeasured anion is the most important requirement in patients presenting with metabolic acidosis and increased anion gap. For this, lactate, 3-hydroxybutyrate, acetoacetate and organic acids are analyzed. The largest group consisting of organic acidemias, including entities such as methylmalonic, propionic and isovaleric acidemias are easily recognized by their typical organic acid profiles. In addition to specific organic acid intermediates, plasma lactate is often elevated in organic acidemias as a result of secondary interference with CoA metabolism. A flowchart for the evaluation of patients with metabolic acidosis with increased anion gap[5] is presented in Figure 4.

Metabolic acidosis with a normal anion gap is noted in renal tubular acidosis or diarrhea. A history of diarrhea is usually sufficient to distinguish hyperchloremic metabolic acidosis due to excessive gastrointestinal bicarbonate losses from that arising from renal tubular dysfunction. Metabolic disorders associated with renal tubular acidosis include hereditary fructose intolerance, hepatorenal tyrosinemia, cystinosis, Fanconi-Bickel syndrome, Lowe syndrome, vitamin D dependency and congenital lactic acidosis due to cytochrome c oxidase deficiency.

## Lactate

Lactate and pyruvate are normal metabolites. Their plasma levels depend upon the equilibrium between their cytoplasmic production from glycolysis and their consumption by different tissues. Abnormal accumulation of lactic acid is a common cause of pathological metabolic acidosis. In a majority of cases, it is caused by tissue hypoxia resulting from inadequate oxygen supply or poor circulation. Blood lactate accumulates in circulatory collapse, hypoxic insult and other conditions involving failure of cellular respiration. These conditions should be ruled out before investigating for an inborn error of lactate pyruvate. Ketosis is absent in most hyperlactacidemias secondary to tissue hypoxia, while it is a nearly constant finding in most inherited metabolic disorders except pyruvate dehydrogenase deficiency, glycogen storage disorder type I and fatty acid oxidation defects. Following are the inherited metabolic causes of lactic acidosis:

1. Defects of pyruvate metabolism
  Pyruvate dehydrogenase deficiency
  Pyruvate carboxylase deficiency
2. Defects of NADH oxidation
  Mitochondrial electron transfer chain defects
3. Disorders of gluconeogenesis/glycogen storage disorders
  Glucose 6-phosphatase deficiency (GSD I)
  Fructose 1,6- bisphosphatase deficiency
  Phosphoenolpyruvate carboxykinase deficiency
  Glycogen debrancher deficiency (GSD III)
  Glycogen synthase deficiency (GSD 0)
4. Fatty acid oxidation defects
5. Defects of biotin metabolism
  Biotinidase deficiency
  Holocarboxylase synthase deficiency
6. Defects of organic acid metabolism
  Propionic acidemia
  Methylmalonic acidemia
7. Others
  Hereditary fructose intolerance

The differential diagnosis of elevated lactate in neurometabolic disorders is shown in Table 6.[31]

**Table 6:** Biochemical clues to differential diagnosis of elevated lactate levels

Lactate (fasting)	Lactate (after meal)	Lactate/ Pyruvate (fasting)	Ketones	Blood Glucose (fasting)	Disorder
(N−) ↑↑↑	(Rise)	(N−) ↑↑	(↑)- ↑↑	N	Respiratory chain defect
(N−) ↑↑↑	Rise	N	N	N	Pyruvate dehydrogenase deficiency
(N−) ↑↑↑	Fall	(N−) ↑	↑↑	(↓)	Pyruvate carboxylase defect
(N−) ↑↑↑	Rise	N	(N−)↑	↓↓	Gluconeogenesis, GSD I
N	(Rise)	N	↑-↑↑	↓↓	GSD types III, 0
N− ↑	(Fall)	N	↓↓	↓↓	Fatty acid oxidation defects
(N−) ↑↑	(Rise)	(N−) ↑↑	↑-↑↑	↓-↑	Organic acidurias

N: Normal, (): Inconstant, ↑: Increased, ↓: Reduced, GSD I, III and 0: Glycogen storage disorder types I, III and 0

## Ketones

Ketonuria is a physiological finding in many cases of late infancy, childhood and even adolescence. Ketosis, which is not associated with acidosis, hyperlactacidemia or hypoglycemia, is likely to be a normal physiological indication of the nutritional state (fasting, catabolism, vomiting, medium-chain triglyceride-enriched or other ketogenic diets). It may be the cause or consequence of repeated vomiting in infants and children. However, hyperketosis at a level that produces metabolic acidosis is not physiological. Hyperglycemia associated with ketosis indicates the presence of diabetes mellitus.

Ketolytic defects (succinyl-CoA: oxo-acid transferase and 3-ketothiolase deficiencies) can present as moderate ketonuria occurring mainly in the fed state at the end of the day.[32] Severe fasting ketonuria without acidosis is often observed in debrancher, and glycogen synthase deficiencies. In both disorders, hepatomegaly, fasting hypoglycemia and postprandial hyperlactacidemia are observed. Ketosis without acidosis is also observed in ketotic hypoglycemias due to adrenal insufficiency, hyperinsulinemic states at any age and growth hormone deficiency in infancy.

## Ammonium

Hyperammonemia is an important laboratory finding associated with neurometabolic disorders presenting mainly with acute encephalopathy. Normally, plasma ammonium is less than 50 µmol/l (reference range: 15–35 µmol/l); however, it may slightly increase as a result of high protein intake, exercise, or a hemolysed sample. Plasma ammonium levels are also often elevated in patients with severe hepatocellular dysfunction irrespective of the cause, including viral infection, intoxications and infection with urease-positive bacteria, particularly with stasis in the urinary tract, Reye syndrome, valproate therapy and leukemia therapy, including treatment with asparaginase. Following are the inherited metabolic disorders presenting with hyperammonemia:

1. Urea cycle defects
  *N*-Acetylglutamate synthetase (NAGS) deficiency
  Carbamoyl phosphate synthetase I (CPS I) deficiency
  Ornithine transcarbamoylase (OTC) deficiency
  Argininosuccinate synthetase (AS) deficiency
  Argininosuccinate lyase (AL) deficiency
  Arginase deficiency
2. Organic acidemias
  Isovaleric acidemia
  Propionic acidemia
  Methylmalonic acidemia
  Glutaric aciduria type II
  Multiple carboxylase deficiency
  3-Ketothiolase deficiency
3. Congenital lactic acidosis
  Pyruvate dehydrogenase deficiency
  Pyruvate carboxylase deficiency
  Mitochondrial respiratory chain defects
4. Fatty acid oxidation defects
  Medium-chain acyl-CoA dehydrogenase (MCAD) deficiency
  Long-chain acyl-CoA dehydrogenase (LCAD) deficiency
  Systemic carnitine deficiency
5. Dibasic amino acid transport defects
  Lysinuric protein intolerance
  Hyperornithinemia Hyperammonemia Homocitrullinuria (HHH) syndrome
6. Hyperammonemia secondary to hepatic dysfunction
  Tyrosinemia type I
  α_1_-Antitrypsin deficiency
  Galactosemia
  Bile acid synthesis defects
  Respiratory chain defects
7. Others
  Glutamate dehydrogenase deficiency (hyperinsulinemia-hyperammonemia syndrome)

**Figure 5:**
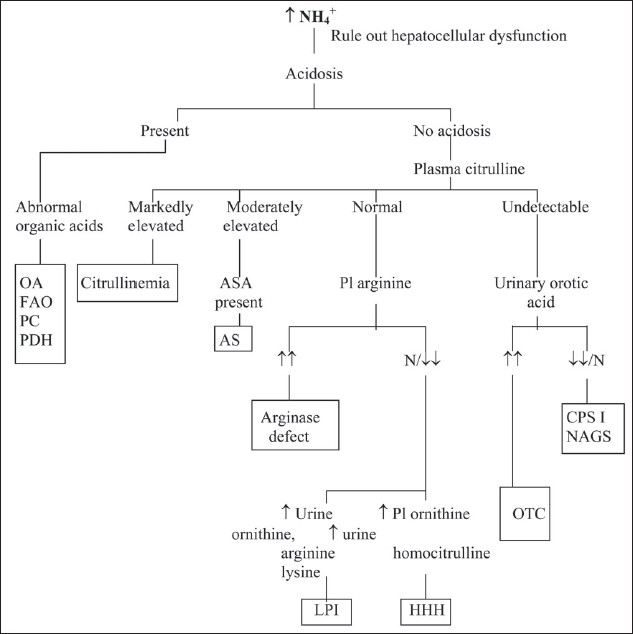
An approach to diagnosis of hyperammonemia in older children OA: organic acidurias, FAO: fatty acid oxidation defects, PC: pyruvate carboxylase deficiency, PDH: pyruvate dehydrogenase deficiency, ASA: argininosuccinic acid, AS: argininosuccinic aciduria, NAGS: N-acetylglutamate synthetase deficiency, CPS I: carbamoyl phosphate synthetase I deficiency, OTC: ornithine transcarbamoylase deficiency, HHH: hyperornithinemia hyperammonemia homocitrullinuria syndrome, LPI: lysinuric protein intolerance

A widely used algorithmic approach to the differential diagnosis of hyperammonemic encephalopathy is presented in Figure 5. A liver function test will help identify whether hyperammonemia is the result of hepatic dysfunction. The presence of moderate to severe metabolic acidosis indicates that hyperammonemia is a disturbance of either organic acid metabolism or β-oxidation of fatty acids or due to congenital lactic acidosis. The most important diagnostic information after ammonium determination, liver function tests and blood gases, is derived from the quantitative analysis of plasma amino acids. The concentration of citrulline is central to the interpretation of the results of amino acid analysis. If the citrulline concentration is markedly elevated, the most probable diagnosis is citrullinemia, resulting from the argininosuccinate synthetase defect. Extremely low citrulline levels suggest a defect in citrulline biosynthesis, which is the result of a deficiency of CPS I, OTC or NAGS. This can be further evaluated by determining the urine orotic acid and orotidine levels. Moderately elevated citrulline levels generally indicate argininosuccinic aciduria, which occurs due to AL deficiency. This disorder is characterized by the presence of markedly elevated argininosuccinate levels in plasma and urine. A normal citrulline concentration with elevated arginine levels is generally sufficiently specific to diagnose arginase deficiency. In typical lysinuric protein intolerence, plasma arginine, ornithine and lysine levels are markedly reduced. At the same time, a marked increase is observed in the excretion of these compounds in urine, along with increased levels of urinary orotic acid and orotidine. In the HHH syndrome, a disorder characterized by a defective transport of ornithine into the mitochondria, plasma ornithine levels are markedly elevated and urinary orotic acid and orotidine levels are increased. In neonates, symptoms of hyperammonemia during the first 24 h after birth observed in a premature infant is suggestive of transient hyperammonemia of the newborn (THAN).

## Amino acids

Analysis of amino acids in various physiological fluids such as plasma, urine and cerebrospinal fluid is central to the investigation of a possible neurometabolic disorder. Some laboratories include a selection of relatively nonspecific chemical tests for the presence of compounds containing certain functional groups such as disulfides by using the cyanide nitroprusside test, etc. However, the presence of large amounts of interfering compounds sometimes makes these methods unreliable. Paper or thin layer chromatography of amino acids in plasma and urine and visualisation by treatment with ninhydrin is useful for detecting excess amino acids such as phenylalanine in phenylketonuria. Quantitative amino acid analysis by ion-exchange chromatography, high-pressure liquid chromatography (HPLC) or tandem mass spectrometry (MS-MS) provides confirmation of the identity and concentrations of the amino acids and provides accurate information on the levels of amino acids that may be present in subnormal concentrations. These methods are also necessary to quantitate reliably the concentration of amino acids in fluids such as CSF.

In inborn errors of specific amino acid metabolism, marked increases are observed in plasma and urine, which are sufficiently specific to suggest a diagnosis. Secondary abnormalities of amino acid concentrations in plasma and urine are also very common. Severe hepatocellular disease, renal tubular disease, catabolic states, malnutrition, malignancy, infections, pregnancy, vitamin deficiencies, burns and other injuries are all associated with the disturbances of amino acid concentrations in plasma, urine or both. Increased amino acid levels in the urine in the absence of corresponding increases in plasma levels occur generally due to inherited or acquired renal transport defects. An important point to be considered is that the concentrations of various amino acids in plasma depend on the metabolic state of the individual. During the postprandial period, the levels of essential amino acids, phenylalanine, tyrosine, lysine, valine, leucine, isoleucine, etc, are increased. Prolonged fasting will result in an elevation of the branched-chain amino acids, leucine, isoleucine and valine. The reference values are based on plasma collected 4–6 h after the last meal.

## Organic acids

Organic acids comprise key metabolites of virtually all pathways of intermediary metabolism as well as exogenous compounds. They are generally determined in urine by the gas chromatography-mass spectrometry (GC-MS), although diagnostically important organic acid changes occur in a variety of physiological fluids. Preparation of the analysis includes preliminary extraction of the organic acids from urine into an organic solvent; this is followed by derivatization to form trimethysilyl derivatives to lower the temperature at which they vaporise and to prevent them from undergoing thermal decomposition, before injecting into the GC-MS. The preliminary identification of the organic acids is based on the retention time, that is, the time taken by the compound to pass through the column. The interpretation of organic acid analysis depends on several key diagnostic metabolites as well as the characteristic patterns of abnormalities.

## Mucopolysaccharides

Urinary excretion of mucopolysaccharides is typically increased in the case of mucopolysaccharidoses, GM_1_ gangliosidosis and multiple sulfatase deficiency.

Excess acidic mucopolysaccharides in urine can be detected and quantified by measuring the change in color of metachromatic dyes such as Alcian blue, dimethylmethylene blue or toluidine blue. Although qualitative spot tests, such as the Berry spot test, are rapid and inexpensive, they produce many false-positive and false-negative results. Uni- or bidirectional electrophoresis or thin layer chromatography of urinary mucopolysaccharides provides information that is important for the classification of the disorders by indicating the relative proportions of specific mucopolysaccharides.

## Oligosaccharides

The oligosaccharides in urine are produced by the incomplete breakdown of the carbohydrate side chains of complex glycoproteins. Analysis of urine oligosaccharides is an inexpensive, although not very specific, screening test for glycoproteinoses. The most widely used method is thin layer chromatography of diluted urine. Increased amounts of free sialic acid are excreted in the case of sialic acid storage disorders and can be determined in urine by spectrophotometry.

## Acylcarnitine and acylglycines

Analysis of carnitine and glycine esters has become an important part of the investigation of organic acidopathies and disorders of mitochondrial β-oxidation. One of the most important advances in the application of diagnostic laboratory technology has been the introduction of MS-MS for the assay of carnitine profile, which allows the detection of many defects of mitochondrial β-oxidation. Compounds eluted from dried blood spots can be used for analyzing acylcarnitines.

Measuring plasma total carnitine is also helpful in determining the presence of a fatty acid oxidation disorder. All but one is associated with either an increased or decreased concentration of total carnitine in the plasma and tissues. In carnitine transporter defect, plasma carnitine levels are severely decreased (<5% of the normal level). In carnitine palmitoyl transferase-1 (CPT-I) deficiency, total carnitine levels are increased (150–200% of the normal level). In all other defects of fatty acid β-oxidation, total carnitine levels are reduced to 25–50%.

## Very-long-chain fatty acids (VLCFA)

The quantitative analysis of the very-long-chain fatty acids (VLCFA) is required for the differential diagnosis of various peroxisomal disorders. The characteristic biochemical abnormalities in some of these disorders are summarized in Table 7. The estimation of VLCFA and phytanic acid requires preliminary extraction and derivatization of complex lipids, followed by thin-layer chromatographic isolation and capillary gas chromatographic analysis of the fatty acid methyl esters.

**Table 7:** Biochemical abnormalities found in some peroxisomal disorders[3]

Disorder	Plasma VLFCA	Urinary pipecolic acid	Plasma phytanic acid	RBC plasmalogens	Plasma bile acid metabolites
Zellweger syndrome	↑↑↑	↑↑↑	↑	↓↓↓	↑↑↑
Neonatal adrenoleukodystrophy	↑↑↑	↑↑	↑	↓↓	↑↑↑
Ketoacyl-CoA thiolase deficiency	↑↑↑	↑↑↑	--	--	↑↑↑
Rhizomelic chondro dysplasia punctata	--	--	↑↑	↓↓↓	--
X-linked adrenoleuko dystrophy	↑↑	--	--	--	--
Adult Refsum disease	--	--	↑↑↑	--	--

## Red Cell Plasmalogens

Plasmalogens are the major components of the phospholipids of myelin and other membranes, including the red blood corpuscles. In peroxisomal biogenesis disorders, a deficiency of plasmalogen biosynthesis occurs, which results in decreased concentrations in myelin and other membranes, including red cell membranes. Plasmalogens are determined by extracting membrane lipids and determining lipid phosphorus after saponification to remove phosphoglycerides.

## Pipecolic acid

Elevated levels of pipecolic acid, an intermediate in lysine metabolism, in the plasma and urine is characteristic of inherited defects of peroxisomal biogenesis. Pipecolic acid is usually determined by HPLC, gas chromatography or GC-MS. Plasma levels may also be elevated in children with hepatocellular disease or by ingestion of vegetables rich in pipecolate.

## Enzyme studies

The final diagnosis of many neurometabolic disorders depends on the ability to demonstrate a specific enzyme deficiency responsible for the disease. Prenatal diagnosis, in particular, requires access to specific enzyme assay in cases in which the diagnosis of the disease under investigation is established. Enzyme analysis can also be used for the detection of carriers. Table 8 shows a summary of the enzyme defects in lysosomal storage disorders and the most accessible source of enzymes for diagnostic analysis. Although fibroblasts are considered the most optimal material for the diagnosis of lysosomal disorders, analysis of the enzyme deficiencies in leukocytes are equally reliable in most cases.

**Table 8:** Enzymes useful in the investigation of lysosomal storage disorders

Disorder	Enzyme	Enzyme source
**Sphingolipidoses**		
GM1 gangliosidosis	β-Galactosidase	S, L, F
GM2 gangliosidoses		S, L, F
Tay Sach disease	β-Hexosaminidase A	S, L, F
Sandhoff disease	β-Hexosaminidase A and B	S, L, F
Metachromatic leukodystrophy	Arylsulfatase A	L, F
Krabbe globoid cell leukodystrophy	Galactocerebrosidase	L, F
Fabry disease	α-Galactosidase A	S, L, F
Gaucher disease	Glucocerebrosidase (β-glucosidase)	L, F
Niemann-Pick disease, types A and B	Sphingomyelinase	L, F
Farber lipogranulomatosis	Ceramidase	L, F
**Mucopolysaccharidoses**		
Hurler disease (MPS I-H)	α-L-iduronidase	S, L, F
Scheie disease (MPS I-S)	α-L-iduronidase	L, F
MPS I variants (MPS IH/S)	α-L-iduronidase	L, F
Hunter disease (MPS II)	Iduronate 2-sulfatase	L, F
Sanfilippo disease type A (MPS IIIA)	Heparan N-sulfatase	L, F
Sanfilippo disease type B (MPS IIIB)	α-N-Acetylglucosaminidase	L, F
Sanfilippo disease type C (MPS IIIC)	Acetyl-CoA: α-glucosaminide acetyl transferase	L, F
Sanfilippo disease type D (MPS IIID)	N-Acetylglucosamine-6-sulfatase	L, F
Morquio disease type A (MPS IVA)	N-Acetyl galactosamine-6-sulfatase	L, F
Morquio disease type B (MPS IVB)	β-Galactosidase	L, F
Maroteaux-Lamy disease (MPS VI)	N-Acetylgalactosamine 4-sulfatase	L, F
Sly syndrome (MPS VII)	β-Glucuronidase	S, L, F
Mucopolysaccharidosis type IX (MPS IX)	Hyaluronidase	S
**Glycoproteinoses**		
α-Fucosidosis	α-Fucosidase	S, L, F
α-Mannosidosis	α-Mannosidase	L, F
β-Mannosidosis	β-Mannosidase	L, F
Sialidosis type I	α-Neuraminidase	L, F
Galactosialidosis	α-Neuraminidase and β-Galactosidase	L, F
Schindler disease	α-N-acetylgalactosaminidase	
Aspartylglucosaminuria	Aspartylglucosaminidase	L, F
I-cell disease and Pseudo-Hurler	All lysosomal enzymes elevated in	L, F
Polydystrophy (Mucolipidosis II and III)	plasma except β-glucosidase	S, F
**Other storage disorders**		
Wolman disease	Acid esterase	L, F
Neuronal ceroid lipofuscinosis 1	Palmitoyl protein thioesterase	L, F
Neuronal ceroid lipofuscinosis 2	Tripeptidyl peptidase I	L, F
Pompe disease	Acid maltase	muscle

## Tandem mass spectrometry

Tandem mass spectrometry (Tandem MS or MS-MS) is a major technological advance in the screening for inherited metabolic diseases.[33] It has the potential for simultaneous and robust multiple disease screening using a single analytical technique. It permits the expansion of screening of many disorders of organic and amino acid metabolism and fatty acid oxidation disorders. Only few spots of blood on a filter paper are required to prepare an adequate specimen. The utility and application of MS-MS has been demonstrated in many countries worldwide.[34,35] This facility is available at NIMHANS, Bangalore and Lal PathLabs, New Delhi.

## Molecular genetic studies

Molecular genetic studies are increasingly relied on to confirm the diagnosis of many neurometabolic diseases. The advent of molecular genetic testing for carrier detection is a major advance in genetic counselling of family members of individuals affected with lysosomal enzyme diseases in which specific disease-causing mutations have been identified. Nonetheless, there remain some limitations on the general diagnostic use of this methodology. With a few exceptions, in all inherited metabolic diseases for which the gene responsible has been isolated and disease-producing mutations characterized, no single mutation accounts for all cases. Patients may have mutant alleles that have not yet been characterized, or they may be so rare that their routine testing may not be economically feasible. Hence, although the detection of a certain mutation in tissue extracted from a patient is considered a strong support for diagnosis, failure to demonstrate the presence of the mutation does not rule out the diagnosis.

## Neuroimaging

**Figure 6:**
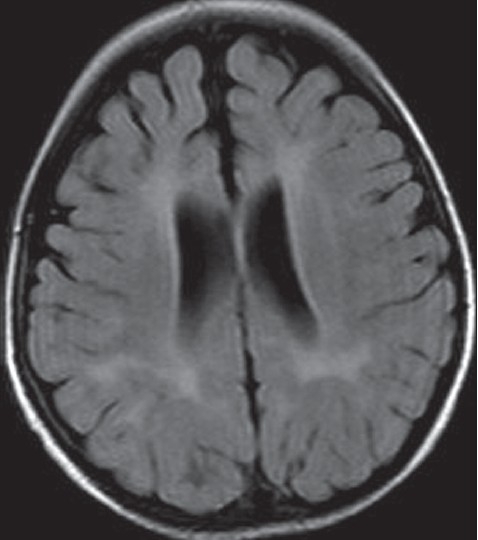
MRI (FLAIR) in a 20 months old girl with phenylketonuria. Note the periventricular hyperintensities

MR imaging has emerged as a powerful tool in the study of normal and abnormal brain structure, function and biochemistry. Clinicians can derive significant benefits from specific imaging findings in neurometabolic disorders.[36] Some of the neurometabolic disorders with typical neuroimaging manifestations are phenylketonuria, maple syrup urine disease, glutaric aciduria type I and methylmalonic acidemia. Although urea cycle disorders are not associated with specific imaging findings, hyperammonemia resulting from these disorders can lead to typical findings and facilitate diagnosis.[37] In classical phenylketonuria, white matter abnormalities are frequently observed. The earliest and most frequent abnormalities are high signal-intensity lesions on T2-weighted images in the parieto-occipital periventricular white matter (periatrial and peritrigonal regions)[38] [Figure 6]. The white matter lesions are symmetrical and either band-like or patchy and partly confluent in an irregular fashion. Frequently, the frontal, and less often, the occipital white matter changes have a peculiar configuration that extends like a small flame from the ventricular border in line with the ventricle.[39]

**Figure 7:**
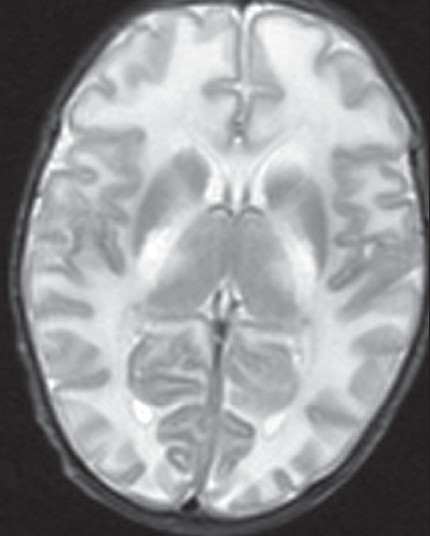
MRI (T2W) in a 14 days- old baby with classic maple syrup urine disease. Note the diffuse symmetrical white matter hyper intensity of white matter. In addition, note involvement of globus pallidi and thalami

In maple syrup urine disease, MRI shows diffuse symmetrical hyperintensities involving the periventricular, deep white matter and subcortical ‘U’ fibers[40] [Figure 7]. The other areas involved are the globus pallidus, thalami and brainstem. The globus pallidus showed diffuse involvement, but the involvement of the thalami was limited mainly to the anterolateral region, sparing the dorsomedial and dorsolateral regions. In the cerebellum, the deep white matter as well as the dentate nuclei shows T2 hyperintensities. In the intermediate form of MSUD, there may be sparing of posterior limb of the internal capsule.[41] Proton MRS is particularly helpful in establishing a diagnosis during metabolic decompensation. A peak is observed at 0.9 PPM, which results from the resonance of the branched-chain amino acids and keto acids.

**Figure 8:**
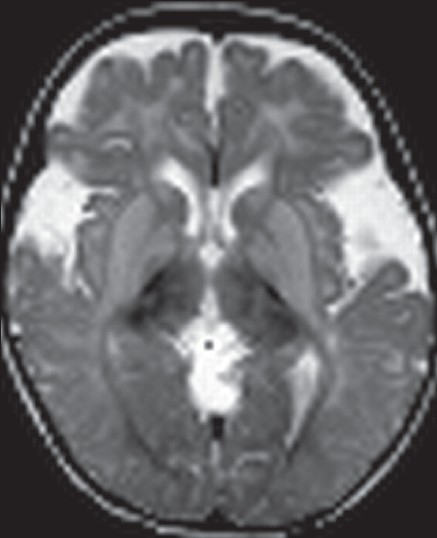
MRI (T2 W) in a 15 months old baby with glutaric aciduria type 1. Note the widened sylvian fissures, bilateral symmetrical signal changes in the basal ganglia. and widened subarachnoid spaces

Organic acidemias are another group of disorders with characteristic neuroimaging findings, which aid in diagnosis. Widening of the sylvian fissure, mesencephalic cistern and expansion of CSF spaces anterior to the temporal lobes are cardinal signs of glutaric aciduria Type-1 [Figure 8]. If combined with abnormalities of the basal ganglia and white matter, glutaric aciduria Type-1 should be strongly suspected.[42] In some patients, delayed myelination or white matter abnormalities are noted.[43,44]

**Figure 9:**
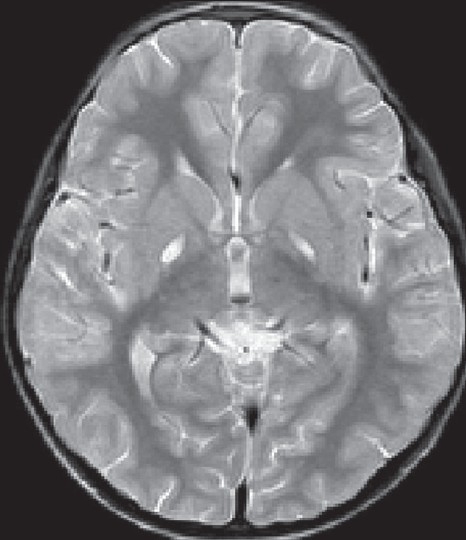
MRI (T2W) in a 12- year old boy with methylmalonic acidemia. Note the bilateral symmetrical hyperintensities involving the medial globus pallidi

The characteristic feature of methylmalonic acidemia is severe involvement of the globus pallidus[45,46] [Figure 9]. The bilateral pallidal necrosis as revealed by neuroimaging occurs due to acute respiratory acidosis with cell necrosis. Involvement of other ganglionic structures is usually not noted. There is no definite explanation for the selective involvement of globus pallidus in methylmalonic acidemia. This finding is noted both in individuals with acute decompensation and in those without such episodes.

## Treatment of inherited neurometabolic disorders

Specific and effective treatment is possible for many inborn errors due to advances in the understanding of their biochemical basis. Early clinical diagnosis is essential in ensuring that the affected infants will be benefited by these advances. Some of the modalities of treatment include regulation of substrate accumulation by restricted dietary intake (phenylalanine in phenylketonuria), regulation of endogenous production of substrate (NTBC treatment of hepatorenal tyrosinemia), acceleration of removal of substrate (dialysis or sodium benzoate in urea cycle disorders), replacement of products (thyroxine in inborn errors of thyroid hormone biosynthesis), enzyme replacement and gene transfer therapy.[47] Treatment of diseases that occur due to mutation in the protein affecting the utilization or binding of a vitamin cofactor, with pharmacological doses of the vitamin, often results in correction of the metabolic defect and reversal of the signs of the disease.

Even when treatment options are limited, there are benefits that follow an early diagnosis. A definitive diagnosis in a sick child avoids further unnecessary investigations, permits an accurate assessment of prognosis, and prevents the loss of an opportunity to make the diagnosis in the case of the death of the child. Furthermore, genetic advice can be offered to families with the prospect of prenatal diagnosis for future pregnancies, identification of other affected family members and carrier detection.

## Conclusion

Diagnosis of neurometabolic disorders always poses difficulty to physicians. This is mainly due to the fact that these disorders are rare and most of them share common clinical manifestations. The main obstacle to making an accurate diagnosis is a failure in considering the possibility clinically. Definitive long-term treatment usually requires that a specific diagnosis is made. With the progress of basic science and technology over the past century, the pathogenesis of these disorders has been understood clearly. This knowledge has opened a possibility of therapeutic intervention for many of these disorders.

## Some useful websites

http://www.mayomedicallaboratories.com

http://www.aruplab.com
